# Anti‐Malondialdehyde Low‐Density Lipoprotein Antibodies and Valvular Calcification: A Substudy of the SCOT‐HEART Trial

**DOI:** 10.1161/JAHA.125.041524

**Published:** 2025-11-03

**Authors:** Adam Hartley, Michelle C. Williams, Amit Kaura, Sarah Verhemel, Mikhail Caga‐Anan, Damini Dey, Marc R. Dweck, Dorian O. Haskard, Michael Joner, Manuel Mayr, David E. Newby, Ramzi Y. Khamis

**Affiliations:** ^1^ National Heart and Lung Institute Imperial College London UK; ^2^ British Heart Foundation Centre for Cardiovascular Science University of Edinburgh UK; ^3^ Biomedical Imaging Research Institute, Department of Biomedical Sciences Cedars‐Sinai Medical Centre Los Angeles CA; ^4^ Technical University of Munich Germany

**Keywords:** anti‐oxidized LDL antibodies, atherosclerosis, coronary CT angiography, oxidized LDL, valvular calcification, Basic Science Research

## Abstract

**Background:**

Coronary computed tomography–derived valve calcification is becoming increasingly important in the multi‐modality assessment of valvular disease, especially in aortic and mitral valve disease. Separately, natural antibodies targeted against malondialdehyde‐modified low‐density lipoprotein (MDA‐LDL), an important subset of all oxidized LDLs, are related to fewer atherosclerosis and cardiovascular events. This study sought to investigate the association between the prevalence of aortic and mitral valve calcification with IgG and IgM anti‐MDA‐LDL antibodies.

**Methods:**

In a substudy of the multicenter randomized controlled SCOT‐HEART trial (Scottish computed tomography of the heart), blood biomarkers were measured using laboratory‐developed enzyme‐linked immunosorbent assays and assessed in tertiles, with adjustment for the ASSIGN cardiovascular risk score.

**Results:**

In 830 patients (53% male, 57.6±9.8 years) with a heavy burden of cardiovascular risk factors, the highest tertile of IgM anti‐MDA‐LDL was associated with a lower prevalence of aortic valve calcification (odds ratio, 0.59 [95% CI, 0.36–0.96], *P*=0.04) and mitral valve calcification (odds ratio, 0.26 [95% CI, 0.07–0.72], *P*=0.02). The highest tertile of IgG anti‐MDA‐LDL was associated with a lower prevalence of mitral valve calcification (odds ratio, 0.36 [95% CI, 0.10–0.99], *P*=0.05), but not aortic calcification.

**Conclusions:**

Anti‐MDA‐LDL antibody levels were associated with a lower prevalence of aortic and mitral valve calcification. Assessment of these novel biomarkers may be useful in screening patients for valve calcification, as well as providing novel insights into potential pathological relationships.

**Nonstandard Abbreviations and Acronyms**
MDA‐LDL: malondialdehyde‐modified low‐density lipoprotein
oxLDL: oxidized LDL
oxPL: oxidized phospholipid
SCOT‐HEART: Scottish computed tomography of the heart

**Clinical Perspective**

**What Is New?**

- Both IgM and IgG anti‐malondialdehyde‐modified low‐density lipoprotein antibody levels are associated with reduced mitral valve calcification, whereas IgM anti‐malondialdehyde‐modified low‐density lipoprotein levels are associated with lower aortic valve calcification.
- These findings suggest that natural antibodies to oxidative epitopes may exert a protective effect against valvular degeneration.

**What Are the Clinical Implications?**

- Anti‐malondialdehyde‐modified low‐density lipoprotein antibodies may serve as blood‐based biomarkers to identify individuals at lower risk of valvular calcification during cardiovascular imaging.

Calcification is one of the hallmarks of advanced atherosclerotic plaques, and while spotty calcification is associated with plaque vulnerability to rupture, more uniform dystrophic calcification may be related to plaque stability. Atherosclerosis can occur within vascular beds of coronary, cerebral, or peripheral arteries, as well as within central blood vessels and cardiac valves.

Atherosclerosis and valvular calcification share common pathological mechanisms. Valvular calcification is influenced by common cardiovascular risk factors and is associated with an increased risk of cardiovascular events.^1^ This process involves oxidized phospholipids (oxPLs), which are generated through pro‐oxidant pathways and act as danger‐associated molecular patterns, triggering inflammatory and calcification pathways.^2^, ^3^ Antibodies targeting oxPLs, such as the murine monoclonal antibody E06, have been shown to inhibit their pro‐inflammatory and pro‐calcific effects, highlighting the protective potential of such immune mediators.^4^, ^5^, ^6^ Circulating oxPLs carried by lipoprotein(a) and measured as oxPL‐apolipoprotein‐B are strongly associated with aortic valve calcification and its progression.^3^, ^7^, ^8^, ^9^, ^10^, ^11^, ^12^, ^13^

Malondialdehyde modification of low‐density lipoprotein (MDA‐LDL) forms a major component of the group of heterogeneous lipid peroxidation–specific epitopes on oxidized LDL (oxLDL) (domains on the oxLDL antigen that the antibodies may bind) and can undergo recognition by naturally occurring antibodies targeted against MDA‐LDL.^9^ These natural antibodies, part of the innate immune system, are preformed, germline‐encoded immunoglobulins that help remove oxLDL and other debris from the body by facilitating their transport to the reticuloendothelial system for disposal.^14^ They offer an immediate, broad defense against potential pathogens. Natural IgM anti‐MDA‐LDL antibodies are generally considered protective and linked to reduced atherosclerotic disease and cardiovascular events; however, the role of IgG anti‐MDA‐LDL antibodies is still under investigation.^15^, ^16^, ^17^, ^18^, ^19^

While oxLDL is known to play a role in atherosclerosis and vascular calcification, its role in valvular calcification may involve immune responses to oxidation‐specific epitopes, including the production of IgM and IgG antibodies to MDA‐LDL.^16^, ^20^ These antibodies may modulate inflammatory and osteogenic responses within valve tissue, but this remains poorly understood.

Therefore, antibodies targeting oxidized lipid epitopes, including MDA‐LDL and oxPLs, may play a critical role in modulating inflammatory and calcification processes in vascular and valvular diseases. Furthermore, such antibodies could serve as biomarkers or therapeutic targets for conditions such as calcific aortic valve disease.

This substudy of the SCOT‐HEART (Scottish computed tomography of the heart) trial investigates whether coronary computerized tomography angiography (CCTA)–derived measures of cardiac valvular calcification are associated with anti‐MDA‐LDL antibodies.

## METHODS

The data that support the findings of this study are available from the corresponding author upon reasonable request.

### Study Design

The SCOT‐HEART study was a multicenter prospective open‐label randomized controlled trial that assessed the role of CCTA in patients with suspected angina due to coronary heart disease (ClinicalTrials.gov. identifier: NCT01149590). The study was approved by the local ethics committee, and written informed consent was provided by all participants. The study design has been described previously and the primary findings reported.^21^, ^22^, ^23^ Briefly, participants were recruited from specialist cardiology chest pain clinics and underwent standard of care investigations as per local clinical protocols, including stress electrocardiography, stress imaging, and invasive coronary angiography, and were randomized to standard of care alone or standard of care plus CCTA. A total of 4146 patients aged 18 to 75 were recruited, of which 2073 underwent randomization to the CT arm.^21^ Blood samples were available in 830 of these patients, which therefore comprised the population for this substudy. All imaging and biomarker data used in this analysis were collected at baseline, and all analyses were performed using a cross‐sectional design. The 10‐year cardiovascular risk was provided by the ASSIGN (assessing cardiovascular risk using SIGN guidelines) score. This cardiovascular risk score identifies people who are likely to develop cardiovascular disease over the following 10 years, including risk from social deprivation. Importantly, it has been calibrated to the Scottish population and performs better than the Framingham score at risk discrimination in Scotland.^24^, ^25^

### Coronary Computed Tomography Angiography

Participants underwent both noncontrast CT and contrast‐enhanced CCTA. Imaging was performed at the Edinburgh Imaging facility using a 320‐detector row scanner (Aquilion ONE, Toshiba Medical Systems, Japan). Tube voltage, current, and contrast volume were adjusted based on body mass index.

CCTA images were assessed by at least 2 trained observers. Aortic and mitral valve calcification was visually assessed as being either present or absent.

### MDA‐LDL Antibody Biomarker Assessment

Blood specimens were obtained at the time of intravenous cannulation for CT scanning and stored at −80 °C. Laboratory non‐commercially developed enzyme‐linked immunosorbent assays were used to measure all serological biomarkers using the blood serum fraction, which have been extensively validated previously.^18^, ^19^, ^26^, ^27^, ^28^

Serum samples were assessed for IgG and IgM anti‐MDA‐LDL concentrations, in indirect enzyme‐linked immunosorbent assay format. MDA‐LDL (10 μg/mL) was used to coat for IgG/IgM anti‐MDA‐LDL antibodies; the production of which is described below. The primary detection antibodies were unlabeled mouse anti‐human IgG (Southern Biotech, Birmingham, AL, 1:2000) or biotinylated mouse anti‐human IgM (Southern Biotech, Birmingham, AL, 1:2000) for IgG and IgM anti‐MDA‐LDL respectively. The secondary detection antibodies were horseradish peroxidase–conjugated streptavidin (R&D Systems, Minneapolis, MN, 1:200) for IgM anti‐MDA‐LDL and horseradish peroxidase–conjugated polyclonal rabbit anti‐mouse antibody (Dako, Cambridgeshire, UK, 1:2000) for IgG anti‐MDA‐LDL. 3,3′,5,5′‐tetramethylbenzidine (Sigma Aldrich, Poole, UK) was added as substrate, before stopping the reaction with 0.5 mol/L H_2_SO_4_. Plates were read at an optical density of 450 nm using a Synergy HT microplate reader (BioTek, VT).

Acid hydrolysis of malondialdehyde bis (dimethyl acetal) (Sigma‐Aldrich, Poole, UK) was performed to produce 0.5 mol/L MDA solution, which was subsequently incubated with native LDL at 37 °C for 3 hours, as performed previously.^29^ This generated MDA‐LDL, which was then eluted through a Zeba Spin desalting column (ThermoFisher Scientific, Waltham, MA), and phosphate‐buffered saline/0.01% EDTA added to prevent additional oxidation.

A standard curve from reference serum was used to correct sample biomarker values per enzyme‐linked immunosorbent assay run after subtraction of background signal. Raw optical density values were used, expressed as absorbance units at 450 nm. Intraplate and interplate coefficients of variance were <5% and <15%, respectively. All analyses were undertaken by staff blinded to patient characteristics.

### Statistical Analysis

Continuous variables are presented as mean±SD or median with interquartile range, depending on the normality of the distribution. Categorical variables are presented as numbers with percentages. Statistical analysis was performed using R version 4.0.3 (R Foundation for Statistical Computing, Vienna, Austria). Measured biomarkers were divided into tertiles with the lowest tertile used as reference, as performed previously.^19^ A binary logistic regression model was used to evaluate the association between antibody concentrations and valve calcification, adjusted for cardiovascular risk score, which was modeled using a restricted cubic spline with 3 knots.

Splines were used in the regression model because the assumption of linearity between antibody concentration and valve calcification was not satisfied. The splines transform the explanatory variable (antibody concentration) by splitting the range of values in intervals and fitting a separate curve in each interval. The spline is defined so that the overall resulting curve is smooth and continuous. The points that delimit the intervals are called knots. Preliminary investigation suggested that 3 knots should be used to model antibody concentrations in the restricted cubic spline analyses. The rms package defaults to placing 3 knots for restricted cubic splines at the 10th, 50th, and 90th percentiles of the predictor variable, because this configuration provides a practical balance between model flexibility and stability.^30^ This choice allows the model to capture potential nonlinearity in the center of the data (via the median knot) while accommodating curvature in the tails without overfitting. It also ensures robustness to outliers, because extreme values beyond the 10th and 90th percentiles have limited influence. This default follows earlier recommendations, emphasizing that knots should be placed to reflect the distribution of the data while avoiding sensitivity to sparse or extreme regions. A statistically significant difference was defined as a 2‐sided *P* value <0.05.

## RESULTS

### Patient Population

This study included all 830 patients enrolled in the SCOT‐HEART trial who had undergone CCTA and had serum available for analysis (Table 1). The mean age of the patients was 58±10 years. There was a high prevalence of cardiovascular risk factors, whereas only a few had been diagnosed with prior coronary heart disease. The study population was representative of the overall SCOT‐HEART population (Tables S1 and S2).

**Table 1:** Patient Characteristics for Included Participants

	All participants
Number	830
Male, n (%)	438 (53)
Age, y (mean±SD)	57.6±9.8
Body mass index, kg/m^2^ (mean±SD)	29.6±5.6
Atrial fibrillation, n (%)	16 (1.9)
Previous coronary heart disease, n (%)	67 (8.1)
Previous cerebrovascular disease, n (%)	40 (4.8)
Previous peripheral vascular disease, n (%)	15 (1.8)
Smoking habit, n (%)	
Current smoker	165 (20)
Ex‐smoker	291 (35)
Nonsmoker	374 (45)
Hypertension, n (%)	297 (36)
Diabetes, n (%)	96 (12)
Family history of coronary heart disease, n (%)	341 (42)
Chest pain diagnosis, n (%)	
Atypical angina	207 (25)
Nonanginal	283 (34)
Typical angina	340 (41)
ASSIGN cardiovascular risk score (median, IQR)	16 (9, 24)

ASSIGN indicates assessing cardiovascular risk using SIGN guidelines; and IQR, interquartile range.

The median IgG anti‐MDA‐LDL was 0.932 [0.702–1.234] absorbance units whereas IgM anti‐MDA‐LDL was 1.614 [1.147–2.211] absorbance units.

### Clinical Characteristics

While there were no associations between clinical characteristics and IgG anti‐MDA‐LDL antibodies (Table S3), IgM anti‐MDA‐LDL antibodies were associated with a range of clinical variables (Table S4). Across tertiles from low‐to‐high, there were inverse relationships with male sex (62% versus 42%, *P* <0.001), cerebrovascular disease (7.2% versus 2.5%, *P*=0.036), hypertension (42% versus 28%, *P*=0.001), diabetes (16% versus 8%, *P*=0.02), and cardiovascular risk score (19.8±11.9 versus 17.5±11.0, *P*=0.011).

### Valvular Calcification

#### IgG Anti‐MDA‐LDL

**Figure 1:**
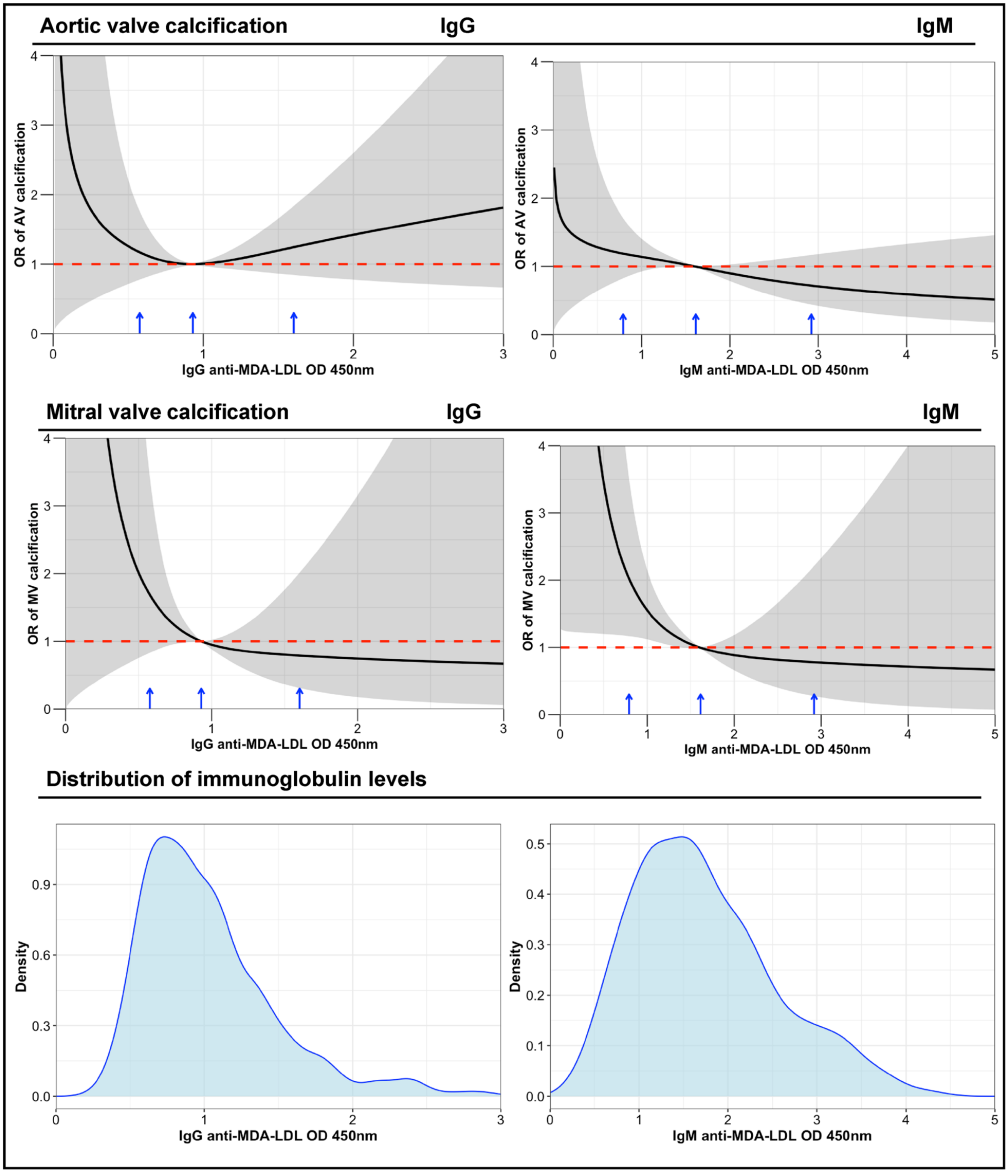
Adjusted association between antibody level and presence of calcification in the aortic valve and mitral valve using restricted cubic splines. Splines were used in the model because the assumption of linearity between antibody concentration and cardiac calcification was not satisfied. Splines were adjusted for cardiovascular risk score. Preliminary investigation suggested that 3 knots (intervals) should be used to fit the spline curve. The locations of the 3 knots are indicated by the blue arrows on the *x*‐axis. The shaded area around the spline curve represents the 95% CI. The probability density distribution is displayed to describe the distribution of measurements of antibody levels to model antibody concentrations in the restricted cubic spline analyses. AV indicates aortic valve; MDA‐LDL, malondialdehyde‐modified low‐density lipoprotein; MV, mitral valve; OD, optical density; and OR, odds ratio.

There was no association between IgG anti‐MDA‐LDL levels and aortic valve calcification. However, the highest tertile of IgG anti‐MDA‐LDL was associated with a lower prevalence of mitral valve calcification (odds ratio, 0.36 [95% CI, 0.10–0.99], *P*=0.05) (Table 2, Figure 1).

**Table 2:** Adjusted Odds Ratio for the Presence of Vascular or Valvular Calcification Per Tertile of IgG/IgM Anti‐MDA‐LDL Antibodies

	Aortic valve calcification		Mitral valve calcification	
	OR (95% CI)	*P* value	OR (95% CI)	*P* value
IgG anti‐MDA‐LDL OD 450 nm (AU)				
Lowest (0.26–0.78)	1.00 (Ref)		1.00 (Ref)	
Middle (0.79–1.11)	0.91 (0.55–1.49)	0.70	0.61 (0.23–1.53)	0.30
Highest (1.12–2.96)	0.99 (0.61–1.62)	0.98	0.36 (0.10–0.99)	0.048*
Trend		0.46		0.25
IgM anti‐MDA‐LDL OD 450 nm (AU)				
Lowest (0.20–1.27)	1.00 (Ref)		1.00 (Ref)	
Middle (1.28–2.00)	0.78 (0.48–1.26)	0.32	0.35 (0.11–0.92)	0.047*
Highest (2.01–4.40)	0.59 (0.36–0.96)	0.04*	0.26 (0.07–0.72)	0.02*
Trend		0.07		0.049*

MDA‐LDL indicates malondialdehyde‐modified low‐density lipoprotein; and MDA‐OD, optical density in AU (absorbance units).

* Significant *P* value <0.05; *P* values based on a logistic regression model across the 3 tertiles of the antibodies with adjustment for cardiovascular risk score. Cardiovascular risk score was modeled using a restricted cubic spline with 3 knots.

#### IgM Anti‐MDA‐LDL

Higher levels of IgM anti‐MDA‐LDL antibodies were associated with a lower prevalence of aortic valve calcification (highest tertile odds ratio, 0.59 [95% CI, 0.36–0.96], *P*=0.04). Graphically, this relationship demonstrated a gradual linear decline in calcification with increasing IgM levels. Similarly, reductions in mitral valve calcification were observed in the middle (odds ratio, 0.35 [95% CI, 0.11–0.92], *P*=0.05) and highest (odds ratio, 0.26 [95% CI, 0.07–0.72], *P*=0.02) tertiles of IgM anti‐MDA‐LDL (Table 2, Figure 1).

## DISCUSSION

In this large SCOT‐HEART substudy, we found that patients with higher circulating anti–MDA‐LDL antibody levels (particularly IgM) had a significantly lower prevalence of both aortic and mitral valve calcification. IgG anti–MDA‐LDL also showed a protective trend for aortic valve calcification (with the highest IgG tertile demonstrating lower odds of aortic calcification), although this did not reach statistical significance for mitral valve calcification. To our knowledge, this is the first study to demonstrate an inverse association between oxidation‐specific antibodies and valvular calcification, highlighting a novel link between humoral immunity to modified LDL and the pathogenesis of calcific valve disease. These findings align with growing evidence that immune responses to oxidized lipids can modulate cardiovascular disease. Natural antibodies against oxLDL (such as those targeting MDA‐LDL adducts) have been associated with attenuated atherosclerosis and freedom from cardiovascular events in prior studies. Indeed, in our own cohort and others, higher IgM anti–oxLDL levels have correlated with a lower burden of coronary atheroma and more stable plaque characteristics.^18^, ^19^, ^31^ Our results extend this paradigm to valvular disease, suggesting that similar protective immunity against oxLDL that slows coronary plaque progression may also mitigate calcific degeneration in the aortic and mitral valves. By demonstrating that individuals with robust anti–MDA‐LDL responses tend to have less valve calcification on CT, we provide further evidence of an immune–cardiovascular axis linking atherosclerosis and calcific valve sclerosis.

### Potential Mechanistic Role of Anti‐MDA‐LDL Antibodies in Valvular Calcification

Insights from animal and human tissue analyses highlight the multifaceted pathways contributing to calcific aortic valve disease.^32^, ^33^, ^34^, ^35^ Inflammation, lipid oxidation, neurohormonal signaling, and osteogenic differentiation drive the progressive fibro‐calcific transformation of valve tissue. Multi‐omics profiling has further elucidated gene regulatory networks implicated in disease progression.^36^ However, the link between these molecular drivers and clinical calcification remains incompletely understood.

OxLDL, particularly MDA‐LDL, contributes to chronic inflammation and mineralization in valve leaflets.^37^ Once oxidized, LDL particles expose neoepitopes that act as danger signals, triggering immune responses. Natural IgM antibodies, part of the innate immune repertoire, recognize such epitopes and promote their clearance via opsonization and phagocytosis.^14^, ^38^ This may reduce local oxidative stress and prevent downstream inflammatory activation, including the transformation of valvular interstitial cells into osteoblast‐like phenotypes. In our study, higher IgM anti‐MDA‐LDL levels were independently associated with reduced prevalence of both aortic and mitral valve calcification, consistent with their known atheroprotective role.^16^, ^20^

IgG antibodies are generated through adaptive immune mechanisms and exhibit more heterogeneous effects. While some IgG subclasses may assist in debris clearance and immune complex formation, others may propagate inflammation via Fc receptor‐mediated pathways. Our observation of a protective association between IgG anti‐MDA‐LDL and mitral valve calcification (but not aortic) raises questions regarding isotype‐specific effects and tissue context. Additional studies are needed to clarify these differential immunological responses.

Bhatia et al^39^ have shown that oxPLs, particularly those carried by lipoprotein(a), play a pathogenic role in valvular calcification. OxPLs induce mineralization of valve interstitial cells via lysophosphatidic acid signaling and NF‐kB/interleukin‐6/BMP pathways. We speculate that antibodies against MDA‐LDL may partially mitigate such effects by neutralizing oxidative lipids upstream in this cascade, thus dampening pro‐calcific signaling.

### Mitral Annular Calcification and Aortic Stenosis

Mitral annular calcification and aortic stenosis share overlapping pathophysiological features and frequently coexist.^40^ Calcification of the fibrous mitral annulus may disrupt valvular function and contribute to global myocardial remodeling. Calcification in these key regions not only compromises the function of the mitral valve by stiffening the annulus but may also indirectly impact the aortic valve. This progression of calcification can exacerbate the obstruction observed in aortic stenosis, potentially amplifying the hemodynamic effects on both valves. Given the anatomical and functional continuity of the mitral–aortic complex, immune‐mediated mechanisms driving calcification in 1 valve may influence adjacent structures. This underscores the relevance of studying humoral immunity across multiple valvular sites.

### Clinical Cardiovascular Risk Factors and Anti‐MDA‐LDL Antibodies

We found that IgM anti‐MDA‐LDL antibody levels were inversely related to cardiovascular risk factors such as diabetes, hypertension, and cerebrovascular disease. This may reflect consumption or downregulation of these antibodies in the presence of chronic oxidative stress.^41^, ^42^, ^43^ Prior studies from our group demonstrated that anti‐MDA‐LDL antibody levels decline following major oxidative insults, such as cardiac surgery,^26^, ^44^ further supporting their dynamic role in immune homeostasis.

Our results can be contrasted with those of prior studies in different populations. Notably, a substudy of the ASTRONOMER trial^20^ (which examined patients with mild‐to‐moderate aortic stenosis) reported no significant association between circulating anti‐oxLDL antibody titers and the progression of aortic valve stenosis. There are several possible reasons for this discrepancy. First, the patient populations differ: our study evaluated an unselected stable chest pain cohort (median age ≈58 years old, with mostly subclinical valve calcification), whereas ASTRONOMER focused on younger patients with established early aortic valve disease. It is conceivable that oxLDL antibodies play a greater role in the initiation of valve calcification (as captured by our cross‐sectional presence/absence analysis) than in the propagation of advanced stenosis, where mechanical factors and advanced osteogenic activity may overshadow any immunomodulatory effects. Second, the end points differ; we assessed the presence of any CT‐detectable calcification in valves, whereas ASTRONOMER tracked hemodynamic progression over time. Antibody levels might influence whether calcific foci develop, without necessarily halting growth once calcification is underway. Finally, methodological differences in antibody measurements (eg, targeting of MDA‐LDL specifically, isotype distinctions) and limited sample sizes could affect the ability to detect associations. Despite these differences, both studies underscore the multifactorial nature of calcific valve disease, and they highlight the need for further research (particularly longitudinal studies) to clarify whether high levels of anti–MDA‐LDL antibodies can slow valvular calcium accumulation or delay the onset of clinically significant stenosis.

The clinical implications of these findings are notable. Anti‐MDA‐LDL antibodies may serve as biomarkers of reduced susceptibility to valvular calcification. Measuring these antibodies could inform cardiovascular imaging strategies or refine risk stratification in patients undergoing structural heart assessment. Furthermore, the concept of boosting natural antibody responses (via immunization or passive therapies) represents a novel approach to halting early valvular degeneration. Such immunomodulatory interventions, though speculative, merit exploration.

## LIMITATIONS

The main limitations of this study include its focus on serological biomarkers in relation to cardiac atherosclerosis, even though circulating biomarkers likely reflect systemic atherosclerotic burden. Future studies should explore associations across other vascular territories. Our sample size, while substantial, remains modest for exploring downstream clinical outcomes with sufficient statistical power. Moreover, we have performed correction for a composite cardiovascular risk score, but there remains the possibility of confounding from comorbidities not included in this risk score, such as chronic renal disease. Additionally, because this was an exploratory post‐hoc analysis assessing 2 antibody types (IgG and IgM) across 2 valves (aortic and mitral), there is an inherent risk of false positives due to multiple comparisons. We did not apply formal corrections for multiple testing, because the goal was hypothesis generation rather than definitive inference, and results should be interpreted accordingly. Another limitation lies in the specificity of the biomarkers assessed. As with previous studies on antibodies to oxLDL, we evaluated only 1 oxidation‐specific epitope (MDA‐LDL), leaving open the possibility that other important oxidative modifications may have been missed. Bhatia et al^39^ have emphasized the importance of more granular analysis of oxPLs across different lipoprotein carriers, because variation in oxPL content may influence biological outcomes. Nevertheless, anti‐MDA‐LDL antibodies constitute a major subset of the natural antibody pool against oxLDL.^45^ MDA‐LDL itself is a highly relevant pathological epitope, commonly found in atherosclerotic plaques and already a target of emerging therapeutic approaches.^46^, ^47^ Furthermore, no competition assays, for example, with MDA‐bovine serum albumin or native LDL, were performed; however, prior studies using the assays suggest that only MDA‐modified LDL (and not irrelevant MDA‐modified proteins or unmodified LDL) compete for binding.^26^ While mechanistic conclusions cannot be drawn from this correlative analysis, the possibility that some individuals possess a protective reservoir of antibodies that mitigate global atherogenesis warrants further investigation.^46^, ^48^, ^49^, ^50^, ^51^

## CONCLUSIONS

This SCOT‐HEART substudy demonstrates an inverse association between circulating anti–MDA‐LDL antibodies (particularly IgM) and valvular calcification on coronary CT, implicating humoral immunity in the early pathogenesis of calcific valve disease. These findings support the hypothesis that natural antibodies to oxidation‐specific epitopes may confer protection against valvular degeneration and suggest potential for their use as biomarkers or therapeutic targets. Future prospective studies with serial imaging and mechanistic insight are needed to clarify causality, optimize risk stratification, and explore immunomodulatory strategies for prevention of calcific cardiovascular disease.

## Sources of Funding

The SCOT‐HEART trial was funded by The Chief Scientist Office of the Scottish Government Health and Social Care Directorates (CZH/4/588), with supplementary awards from Edinburgh and Lothian’s Health Foundation Trust and the Heart Diseases Research Fund. All researchers were independent of the trial funders. The Royal Bank of Scotland supported the provision of 320‐multidetector CT for NHS Lothian and the University of Edinburgh. The Clinical Research Imaging Centre (Edinburgh) is supported by the National Health Service Research Scotland (NRS) through National Health Service Lothian Health Board. The Clinical Research Facility Glasgow and Clinical Research Facility Tayside are supported by National Health Service Research Scotland (NRS). The SCOT‐HEART trial was co‐sponsored by the University of Edinburgh and NHS Lothian Health Board. R.Y. Khamis is funded by a British Heart Foundation Clinical Research Fellowship (FS/17/16/32560). A. Hartley is funded by a Wellcome Trust Clinical Research Fellowship (220 572/Z/20/Z). M.C. Williams (FS/ICRF/20/26002) and Dr Newby (CH/09/002, RG/20/10/34966, RE/18/5/34216) are supported by the British Heart Foundation. Dr Newby is the recipient of a Wellcome Trust Senior Investigator Award (WT103782AIA). A. Kaura is funded by a British Heart Foundation clinical research training fellowship (FS/20/18/34972). We also acknowledge funding from the National Institute for Health Research (NIHR) Biomedical Research Centre (BRC) for blood sample analysis. We would also like to thank the Sansour Fund at Imperial College Healthcare Charity for their contribution. The work also was supported by the British Heart Foundation Imperial Centre of Research Excellence Award (RE/24/130023).

## Disclosures

D. Dey received software royalties from Cedars‐Sinai Medical Center. D. Dey holds a patent (US8885905B2 in the United States and WO patent WO2011069120A1, Method and System for Plaque Characterization).

## Supporting information

Tables S1–S4
