# Supplementary material for: Anti‐Malondialdehyde Low‐Density Lipoprotein Antibodies and Valvular Calcification: A Substudy of the SCOT‐HEART Trial
Source: J Am Heart Assoc. 2025 Nov 3;14(21):e041524. doi: 10.1161/JAHA.125.041524 (PMC12684796; doi:10.1161/JAHA.125.041524)
Supplement: Supplementary file 1 — Tables S1–S4 [file JAH3-14-e041524-s001.pdf]

SUPPLEMENTAL MATERIAL

Table S1. Patient characteristics of the total SCOT-HEART population and the substudy population.

	**Overall** (n=4146)	**Not included** (n=3316)	**Study Population** (n=830)	**P value**
Male, n (%)	2325 (56)	1887 (57)	438 (53)	0.035
Age, years (mean ± SD)	57 ±10)	57 ± 10	58 ± 10	0.055
Body mass index, kg/m2 (mean ± SD)	30 ± 6	30 ± 6	30 ± 6	0.400
Atrial fibrillation, n (%)	85 (2)	69 (2)	16 (2)	0.888
Previous coronary heart disease, n (%)	401 (10)	334 (10)	67 (8)	0.093
Previous cerebrovascular disease, n (%)	139 (3)	99 (3)	40 (5)	0.012
Previous peripheral vascular disease, n (%)	53 (1)	38 (1)	15 (2)	0.187
Smoking habit, n (%)				0.130
Current smoker	855 (21)	690 (21)	165 (20)	
Ex-smoker	1330 (32)	1039 (31)	291 (35)	
Non-smoker	1954 (47)	1580 (48)	374 (45)	
Hypertension, n (%)	1395 (34)	1098 (33)	297 (36)	0.166
Diabetes Mellitus, n (%)	444 (11)	348 (10)	96 (12)	0.406
Family history of coronary heart disease, n (%)	1716 (42)	1375 (42)	341 (42)	0.935
Chest pain diagnosis, n (%)				<0.001
Atypical angina	988 (24)	781 (24)	207 (25)	
Non-anginal	1692 (41)	1409 (43)	283 (34)	
Typical angina	1462 (35)	1122 (34)	340 (41)	
ASSIGN cardiovascular risk score (median [IQR])	15 [9, 22]	14 [9, 22]	16 [10, 24]	0.002

N=number of patients, %=percentage, SD=standard deviation, kg/m2 =kilograms per square meter, IQR=interquartile range.

Table S2. Patient characteristics of participants who underwent coronary CT angiography in the SCOT-HEART population and the substudy population.

	Overall (n=1778)	Not included (n=948)	Study Population (n=830)	P value
Male, n (%)	997 (56)	559 (60)	438 (53)	0.005
Age, years (mean ± SD)	58 ± 9	58 ± 9	58 ± 10	0.902
Body mass index, kg/m2 (mean ± SD)	30 ± 6	30 ± 5	30 ± 6	0.936
Atrial fibrillation, n (%)	34 (2)	18 (2)	16 (2)	>0.99
Previous coronary heart disease, n (%)	178 (10)	111 (12)	67 (8)	0.011
Previous cerebrovascular disease, n (%)	79 (4)	39 (4)	40 (5)	0.562
Previous peripheral vascular disease, n (%)	31 (2)	16 (2)	15 (2)	>0.99
Smoking habit, n (%)				0.093
Current smoker	330 (19)	165 (18)	165 (20)	
Ex-smoker	593 (34)	302 (32)	291 (35)	
Non-smoker	845 (48)	471 (50)	374 (45)	
Hypertension, n (%)	608 (35)	311 (34)	297 (36)	0.281
Diabetes Mellitus, n (%)	196 (11)	100 (11)	96 (12)	0.591
Family history of coronary heart disease, n (%)	765 (44)	424 (45)	341 (42)	0.130
Chest pain diagnosis, n (%)				0.001
Atypical angina	432 (24)	225 (24)	207 (25)	
Non-anginal	683 (39)	400 (43)	283 (34)	
Typical angina	654 (37)	314 (33)	340 (41)	
ASSIGN cardiovascular risk score (median [IQR])	16 [10, 23]	16 [10, 22]	16 [10, 24]	0.635

N=number of patients, %=percentage, SD=standard deviation, kg/m2 =kilograms per square meter, IQR=interquartile range.

Table S3. Patient characteristics per tertile of IgG anti-MDA-LDL.

	Lowest	Middle	Highest	P value for trend
Male, n (%)	145 (52)	152 (55)	1141 (51)	0.608
Age, years (mean ± SD)	57.2 ± 10	57.2 ± 9.6	58.4 ± 9.9	0.240
Body mass index, kg/m2 (mean ± SD)	29.3 ± 5.7	29.5 ± 5.7	29.9 ± 5.5	0.425
Atrial fibrillation, n (%)	5 (1.8)	7 (2.5)	4 (1.4)	0.636
Previous coronary heart disease, n (%)	26 (9.4)	20 (7.2)	21 (7.6)	0.610
Previous cerebrovascular disease, n (%)	9 (3.3)	16 (5.8)	15 (5.5)	0.315
Previous peripheral vascular disease, n (%)	4 (1.4)	8 (2.9)	3 (1.1)	0.240
Smoking habit, n (%)				0.201
Current smoker	54 (20)	59 (21)	52 (19)	
Ex-smoker	86 (31)	94 (34)	111 (40)	
Non-smoker	137 (50)	123 (45)	114 (41)	
Hypertension, n (%)	103 (38)	93 (34)	101 (37)	0.649
Diabetes Mellitus, n (%)	36 (13)	35 (13)	25 (9)	0.267
Family history of coronary heart disease, n (%)	112 (41)	108 (40)	121 (44)	0.555
Chest pain diagnosis, n (%)				0.510
Atypical angina	74 (27)	65 (24)	68 (25)	
Non-anginal	92 (33)	104 (38)	87 (31)	
Typical angina	111 (40)	107 (39)	122 (44)	
ASSIGN cardiovascular risk score (median, IQR)	15 (10, 23)	16 (9.5, 24)	17 (9, 25)	0.378

N=number of patients, %=percentage, SD=standard deviation, kg/m2 =kilograms per square meter, IQR=interquartile range MDA-LDL=malondialdehyde-modified low-density lipoprotein, IgG=Immunoglobulin G.

Table S4. Patient characteristics per tertile of IgM anti-MDA-LDL.

	Lowest	Middle	Highest	P value for trend
Male, n (%)	172 (62)	149 (54)	117 (42)	<0.001
Age, years (mean ± SD)	58.6 ± 9.5	56.9 ±10.2	57.4 ± 9.7	0.112
Body mass index, kg/m2 (mean ± SD)	29.4 ± 5.1	29.6 ± 6.0	29.8 ± 5.7	0.727
Atrial fibrillation, n (%)	6 (2.2)	7 (2.5)	3 (1.1)	0.434
Previous coronary heart disease, n (%)	25 (9.0)	24 (8.7)	18 (6.5)	0.494
Previous cerebrovascular disease, n (%)	20 (7.2)	13 (4.8)	7 (2.5)	0.036
Previous peripheral vascular disease, n (%)	4 (1.4)	7 (2.5)	4 (1.4)	0.536
Smoking habit, n (%)				0.054
Current smoker	41 (15)	60 (22)	64 (23)	
Ex-smoker	96 (35)	93 (34)	102 (37)	
Non-smoker	140 (51)	123 (45)	111 (40)	
Hypertension, n (%)	116 (42)	105 (39)	76 (28)	0.001
Diabetes Mellitus, n (%)	43 (16)	31 (11)	22 (8)	0.02
Family history of coronary heart disease, n (%)	115 (42)	105 (39)	121 (44)	0.374
Chest pain diagnosis, n (%)				0.07
Atypical angina	66 (24)	72 (26)	69 (25)	
Non-anginal	79 (29)	102 (37)	102 (37)	
Typical angina	132 (48)	102 (37)	106 (39)	
ASSIGN cardiovascular risk score (median, IQR)	17 (11, 26)	15 (8, 23)	14 (9, 24)	0.011

N=number of patients, %=percentage, SD=standard deviation, kg/m2 =kilograms per square meter, IQR=interquartile range, MDA-LDL=malondialdehyde-modified low-density lipoprotein, IgM=Immunoglobulin M.
